# Effects of Adjunctive EDTA and Air-Polishing Powders on Periodontitis-Affected Root Surfaces: A SEM Study

**DOI:** 10.3390/medicina62040753

**Published:** 2026-04-14

**Authors:** Ștefania Sorina Ifrim, Andreea Cândea, Andrada Soancă, Alexandra Roman, Ștefan Vesa, Silviu Albu, Petra Șurlin, Elena Dinte, Emoke Pall, Cosmin Ifrim, Lucian Barbu-Tudoran

**Affiliations:** 1 Department of Periodontology, Applicative Periodontal Regeneration and Pediatrics Dental Medicine Research Center, Iuliu Hațieganu University of Medicine and Pharmacy Cluj-Napoca, Victor Babeș St., No. 15, 400012 Cluj-Napoca, Romania; stefania.sori.hangan@elearn.umfcluj.ro (Ș.S.I.); candea.andreea@umfcluj.ro (A.C.); andrada.popovici@umfcluj.ro (A.S.); 2 Emergency County Clinical Hospital, Clinicilor St., No. 3-5, 400347 Cluj-Napoca, Romania; 3 Department of Pharmacology, Toxicology and Clinical Pharmacology, Iuliu Hațieganu University of Medicine and Pharmacy, Marinescu St., No. 23, 400337 Cluj-Napoca, Romania; 4 2nd Department of Otolaryngology, Iuliu Hațieganu University of Medicine and Pharmacy, Republicii St., No. 18-20, 400015 Cluj-Napoca, Romania; albu.silviu@umfcluj.ro; 5 Department of Periodontology, Research Center of Periodontal-Systemic Interactions, Faculty of Dental Medicine, University of Medicine and Pharmacy of Craiova, 200349 Craiova, Romania; surlinpetra@gmail.com; 6 Department of Pharmaceutical Technology and Biopharmaceutics, Faculty of Pharmacy, Iuliu Hațieganu University of Medicine and Pharmacy, Ion Creanga St., No. 12, 400010 Cluj-Napoca, Romania; edinte@umfcluj.ro; 7 Department of Clinical Sciences, University of Agricultural Sciences and Veterinary Medicine, Calea Manastur 3-5, 400374 Cluj-Napoca, Romania; pallemoke@gmail.com; 8 Department of Prosthetic Dentistry and Dental Materials, Iuliu Hațieganu University of Medicine and Pharmacy Cluj-Napoca, Clinicilor St., No. 32, 400006 Cluj-Napoca, Romania; cosmin.ifrim@elearn.umfcluj.ro; 9 Department of Molecular Biology and Biotechnologies, Faculty of Biology and Geology, Babes-Bolyai University, Clinicilor St., No. 5-7, 400006 Cluj-Napoca, Romania; lucianbarbu@yahoo.com; 10 Electron Microscopy Integrated Laboratory (LIME), National Institute for Research and Development of Isotopic and Molecular Technologies, INCDTIM, 67-103 Donath St., 400293 Cluj-Napoca, Romania

**Keywords:** periodontitis, EDTA, scaling and root planing, air-polishing powders, SEM

## Abstract

***Background***: Mechanical subgingival instrumentation remains the gold standard in periodontitis treatment; however, it may leave residual debris and induce surface alterations. Adjunctive strategies such as air polishing and ethylenediaminetetraacetic acid (EDTA) might optimize root surface conditions. ***Objective***: To evaluate, by scanning electron microscopy (SEM), the effects of scaling and root planing (SRP) combined with EDTA, with or without adjunctive erythritol- or glycine-based air polishing, on root surface alterations and smear layer formation. ***Materials and Methods***: Ten extracted human teeth affected by periodontitis (yielding twenty samples) were included. Two teeth served as descriptive controls. The remaining teeth were allocated to four treatment groups. The first three groups included samples obtained from the middle portion of the roots: S (SRP + EDTA), Se (SRP + erythritol air polishing + EDTA), and Sg (SRP + glycine air polishing + EDTA). The fourth group, Js, consisted of samples from the cementoenamel junction (CEJ) treated with SRP + EDTA. SEM images were appreciated qualitatively and assessed using ordinal scores (0–3) for marks, cracks (×100), and smear layer (×1000). Non-parametric statistics were applied. ***Results****:* A significant difference in mark scores was found among S, Se, and Sg samples (H = 13.411, *p* = 0.001), with Se samples showing lower mark scores than S (*p* = 0.001). Crack scores also differed among groups (H = 12.038, *p* = 0.002), with higher values observed in Se compared to S (*p* = 0.001). Smear layer scores did not differ among groups (H = 0.102, *p* = 0.950). Compared with S samples, Js differed only in marks (*p* = 0.009), with no significant differences in cracks or smear layer. ***Conclusions***: Within the limitations of this in vitro study, root surface alterations and smear layer formation showed variable responses across treatment protocols, with comparable smear layer scores. Similar effects were observed for CEJ and mid-root samples; however, these findings should be interpreted with caution. Further studies are needed to clarify the potential clinical relevance of these observations.

## 1. Introduction

Periodontitis is one of the most prevalent chronic inflammatory diseases worldwide, inducing progressive destruction of the tooth-supporting tissues and resulting in local sequelae, systemic consequences, functional impairment, and reduced quality of life [1,2]. The treatment of periodontitis remains challenging [2,3]. The gold standard of active periodontal therapy is subgingival mechanical instrumentation, also referred to as subgingival debridement or scaling and root planing (SRP) [3,4], which aims to control inflammation [5,6] and improve clinical outcomes [7].

Performing subgingival scaling is technically demanding [3,4] and usually combines hand and ultrasonic instrumentation to enhance procedural efficiency [5,6]. Residual microislands of calculus frequently persist after SRP [8,9,10] due to local or operator-dependent factors [11,12,13] contributing to sustained periodontal inflammation [14,15,16,17,18].

On the other hand, aggressive or repeated subgingival scaling may result in immediate cemental loss and the formation of surface marks [19], or cumulative damage to both hard and soft tissues [20,21,22].

Air-abrasive powders such as glycine and erythritol, applied within the guided biofilm removal concept, have been shown to enhance subgingival biofilm disruption on root surfaces in periodontitis [23], while inducing minimal soft- and hard-tissue damage [24,25,26].

Mechanical SRP of diseased root surfaces, alone or in association with air polishing, may generate residual deposits such as the smear layer [27,28,29], composed of infected cementum, bacterial toxins, biofilm, and calculus remnants [30,31]. The smear layer creates an unfavorable environment for cell attachment [32] during the healing period by acting as a barrier between the root surface and periodontal cells. The amount of smear layer is highly dependent on the subgingival instrumentation approach [19,28,29,33].

Several chemical agents have also been proposed in combination with SRP, including sodium hypochlorite [34], silver nanospheres [35], sulphonic acids [36,37,38], and ethylenediaminetetraacetic acid (EDTA), in order to increase antimicrobial action and promote smear layer elimination [8,11,39]. EDTA is a chelating agent widely used in endodontics due to its ability to bind calcium ions at a neutral pH and remove the inorganic component of the smear layer, thereby exposing the collagen matrix and improving surface permeability [40]. In periodontology, EDTA has attracted attention mostly due to its ability to eliminate residual calculus following SRP [9,10,11,41], improve clot formation and stabilization [42], and exert antibacterial effects [11,43]. Moreover, EDTA application after SRP has been shown to remove the root surface smear layer [9,31] thereby improving cell adhesion on treated areas as a premise for optimal healing [31].

At present, there is still no clear agreement regarding which adjunctive agents or multi-component therapeutic systems offer the greatest benefit in terms of deposit removal and microscopic root surface optimization, which raises the need for new investigations in this area of maximum clinical interest [3,44]. Although the individual effects of SRP, air-polishing powders, and EDTA have been previously described, there is limited in vitro evidence investigating their combined impact on root surface micromorphology.

Based on these considerations, as well as having in view the biological impact of residual subgingival deposits [11,14,15,16,17,18] and the documented adjunctive microscopic effects of EDTA [11,31,44] and air-polishing powders [24,45], the present study aimed to evaluate root surface characteristics in terms of mechanical effects and smear layer accumulation by scanning electron microscopy (SEM) following the use of EDTA in combination with two subgingival air-polishing powders as adjuncts to SRP.

## 2. Materials and Methods

### 2.1. Study Design

The present study evaluated morphological changes in dental root surfaces of extracted human teeth affected by periodontitis following mechanical instrumentation performed with manual and ultrasonic devices or in combination with adjunctive treatments, including air polishing and EDTA application. Root surfaces were qualitatively evaluated by SEM. In addition, post-treatment mechanical damage (instrumentation marks and cracks) and residual surface debris considered as smear layers were semi-quantitatively assessed.

### 2.2. Ethical Considerations

The research protocol was approved by the Ethics Committee of the “Iuliu Hațieganu” University of Medicine and Pharmacy, Cluj-Napoca (Approval No. 160/06 September 2024), and by the Cluj County Emergency Hospital (Approval No. 52951/09 November 2023). Teeth were obtained following extractions performed for periodontal reasons unrelated to the research. Prior to tooth collection, all patients received detailed information about the study protocol and procedures, and written informed consent was obtained from each participant.

All procedures were conducted in accordance with the ethical standards outlined in the Declaration of Helsinki for research involving human subjects, as well as applicable national and European Union regulations.

### 2.3. Sample Selection and Storage

**Figure 1:**
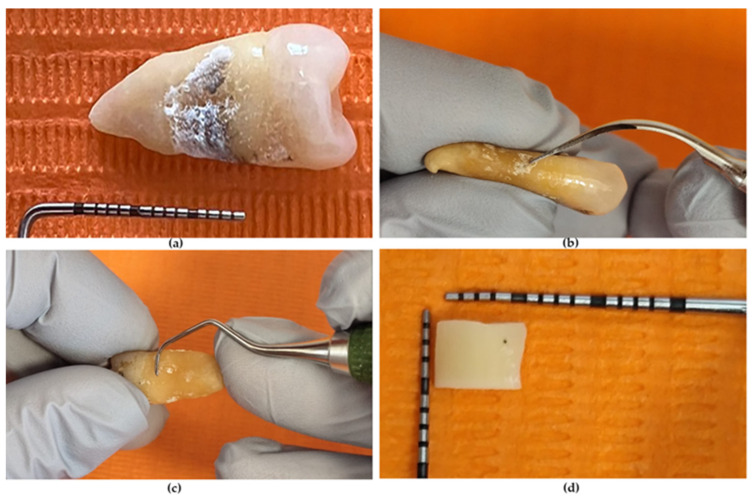
Sample preparation. (**a**) Experimental tooth. (**b**) Ultrasound scaling. (**c**) Manual scaling. (**d**) Root sample.

Ten extracted human teeth (premolars, molars) were selected according to the following inclusion criteria: presence of calculus on the apical two thirds of the root surfaces or near the cementoenamel junction (CEJ) (Figure 1a); absence of root caries; absence of root fractures or anatomical abnormalities; and relatively flat root surfaces allowing sample preparation. Immediately after extraction, teeth were rinsed to remove blood, and soft tissue debris was carefully eliminated. The teeth were stored in 4% chloramine-T solution and used within one month of extraction [29].

### 2.4. Sample Preparation

Each root was marked to outline the area of interest with a 0.7 mm diameter round bur (Komet 801.104.007, Komet, Lemgo, Germany) attached to a high-speed handpiece with water and air cooling. The upper boundary was positioned on the enamel, such that the CEJ was encompassed within the sample, while the apical boundary was traced near the apex. Only external root surfaces were sampled, excluding furcation areas. Special care was taken during sample sectioning and preparation to minimize the risk of mechanical artifacts, including cracks, by using controlled cutting procedures and careful handling of the specimens.

### 2.5. Experimental Groups and Instrumentation Protocol

After marking, the teeth presenting calculus on the apical two thirds of the root surfaces were randomly assigned to one of the treatment groups using a simple random assignment procedure (computer-generated random sequence).

(1) Two teeth were intentionally left uninstrumented to serve as controls;
(2) Two teeth were treated by SRP plus 24% EDTA;
(3) Two teeth were treated by SRP, AIR-FLOW^®^ PERIO Powder (glycine-based) (Electro Medical Systems (EMS), Nyon, Switzerland) and 24% EDTA;
(4) Two teeth were treated by SRP, AIR-FLOW^®^ PLUS Powder (erythritol-based) (EMS, Nyon, Switzerland) and 24% EDTA.

The fifth group was formed by two teeth with calculus on root at CEJ, treated by SRP plus 24% EDTA.

The SRP was carried out using 3.0× surgical loupes and standard clinical protocols. The marked treatment area was initially scaled using an ultrasonic scaler (1S tip, Acteon Group, Paris, France) to remove the visible calculus (Figure 1b). Root surface planing was performed using Gracey curettes no. 5/6 (Hu-Friedy Mfg. Co., LLC, Chicago, IL, USA) (Figure 1c) in the appropriate position, with a modified pen grasp, a working angulation of 45–90° between the cutting edge and the tooth surface, and a stable finger rest. Four strokes were applied on the entire root length using light, standardized operator force to avoid excessive root substance removal [46].

The teeth were subsequently rinsed with water jet unit. The teeth belonging to group 3 and 4 were supplementary treated with erythritol- or glycine-based air-polishing for 5 s from 5 mm working distance [47,48]. All teeth except group 1 received EDTA (Merck KGaA, Darmstadt, Germany) treatment: the samples were immersed in 24% EDTA solution for 2 min, followed by thorough rinsing with water jet unit.

All instrumentations were carried out by a single operator (Ș.S.I.) who had received prior training from an experienced senior (A.R.) to ensure consistency of the instrumentation technique.

#### Sectioning and Sample Identification

Each tooth was sectioned longitudinally (apico-coronal direction) using a tapered fissure diamond bur (1 mm diameter) (Komet 6859.314.010; Komet, Lemgo, Germany) to obtain one section approximately 2 mm in thickness. Special attention was paid, for specimens in group 5, to preserve the CEJ. Subsequently, the most apical portion of the root and the crown were removed, resulting in specimens containing only the treated surfaces.

For SEM evaluation purposes, each specimen was further bisected transversely, producing two samples. The final approximative dimensions of the samples were 4 × 4 × 2 mm (Figure 1d). A total of 20 samples were available. Each tooth was allocated to a single treatment group, and no segments from the same tooth were assigned to different treatments.

The treated surfaces of the root samples were marked at the apical end using a 0.7 mm round bur (Komet 801.104.007; Komet, Lemgo, Germany) to aid SEM orientation. Samples from group 5 were marked at the CEJ. All samples were labeled (Table 1). All samples were stored in distilled water in labeled containers until SEM analysis.

### 2.6. Scanning Electron Microscopy (SEM)

The preparation for SEM analysis involved the following steps: (1) thorough rinsing in sterile water; (2) dehydration through a graded series of ethyl alcohol solutions (ranging from 33% to 100%); (3) mounting of samples on aluminum stubs and storage in a desiccator until SEM examination; (4) sputter coating with approximately 200 Å of gold-palladium immediately before SEM imaging. All samples were examined at various magnifications by a single investigator, who was unaware of the treatment groups.

The samples were scanned at different magnifications (from 100× to 15,000×).

Microscopic evaluations were performed by a senior investigator (A.R.) in collaboration with an experienced SEM operator (L.B.T.) for surface characteristics including cementum presence, exposed collagen fibers and dentin tubuli, instrumentation marks, cracks, residual detritus including smear layer, substance loss, and biofilm-related elements.

Semiquantitative evaluation of post-treatment mechanical damage (instrumentation marks and cracks) and smear layer presence was done by assessing two microscopic fields for each sample, one near the mark and one in the middle of the sample, to capture both instrument-contact regions and general surface characteristics.

The semiquantitative scoring system, adapted from previously reported SEM-based approaches [42,49] and tailored to the objectives of the present study, was defined as follows:

Instrumentation marks (×100):

0 = absent;
1 = few, shallow or isolated lines;
2 = moderate, multiple oriented lines;
3 = heavy, dense, deep dominant pattern.

Surface cracks (×100):

0 = absent;
1 = mild (few, limited);
2 = moderate (clear polygonal network, partial field);
3 = severe (dominant polygonal cracking over most of the field).

Smear layer/residual detritus (×1000):

0 = absent (substrate clearly exposed);
1 = thin, discontinuous layer (mild masking);
2 = moderate detritus (partial to near-complete masking, with limited exposed areas);
3 = heavy layer (complete masking of root surface structures).

Eight evaluations for each group for each parameter were available for statistical analysis.

### 2.7. Statistics

Because the SEM scores were ordinal (0–3) and showed a non-normal distribution, data were summarized using median (25th–75th percentile). Comparisons between S, Se, and Sg were performed using the Kruskal–Wallis test, followed by Bonferroni-adjusted pairwise comparisons. Comparisons between S and Js were performed using the Mann–Whitney U test. A *p* value < 0.05 was considered statistically significant. Given the exploratory nature of the study, a formal statistical power calculation was not performed. The limited sample size should be taken into account when interpreting the findings.

## 3. Results

**Figure 2:**
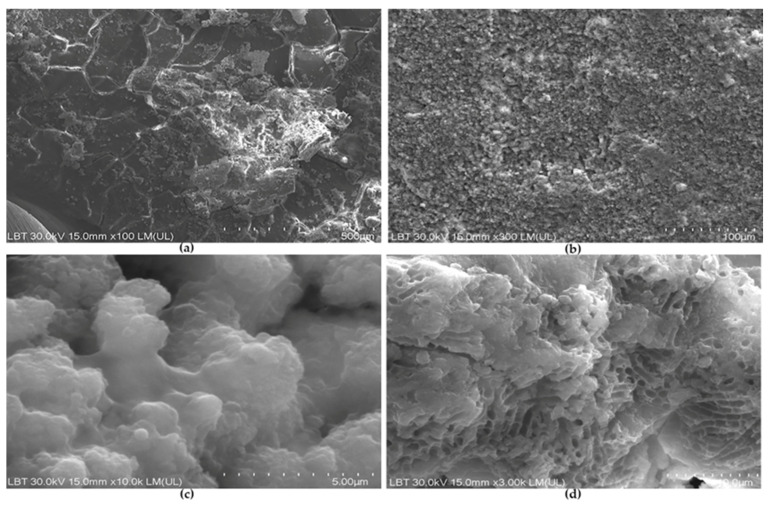
Control root sample (Ct) from middle zone consistent with cementum: (**a**) Calculus-like elevated deposit and some small mineral-like irregularities (×100, 500 µm bar). (**b**) Irregular surface of calculus (×300, 100 µm bar). (**c**) Irregular, fused micro-clusters of calculus with “cauliflower-like” texture (×10.0 k, 5 µm bar). (**d**) Porous honeycomb-like layer relevant for the extracellular polymeric matrix of the biofilm associated with calculus (×3.00 k, 10 µm bar).

SEM observations revealed that Ct samples exhibited extensive dehydration-related polygonal cracking consistent with cementum, some of which presented distinct elevated deposits compatible with calculus (Figure 2a). Calculus deposits exhibited a rough, highly granular surface (Figure 2b), which at higher magnification appeared to be composed of irregular clusters with a rough texture of small agglomerated micronodules (“cauliflower-like” texture) characteristic of mature calculus, with small interparticle spaces (Figure 2c). Some Ct samples, at higher magnification, displayed a porous, honeycomb-like network consistent with remnants of the extracellular polymeric substances (EPS) of the biofilm, largely depleted of intact bacterial cells (Figure 2d).

**Figure 3:**
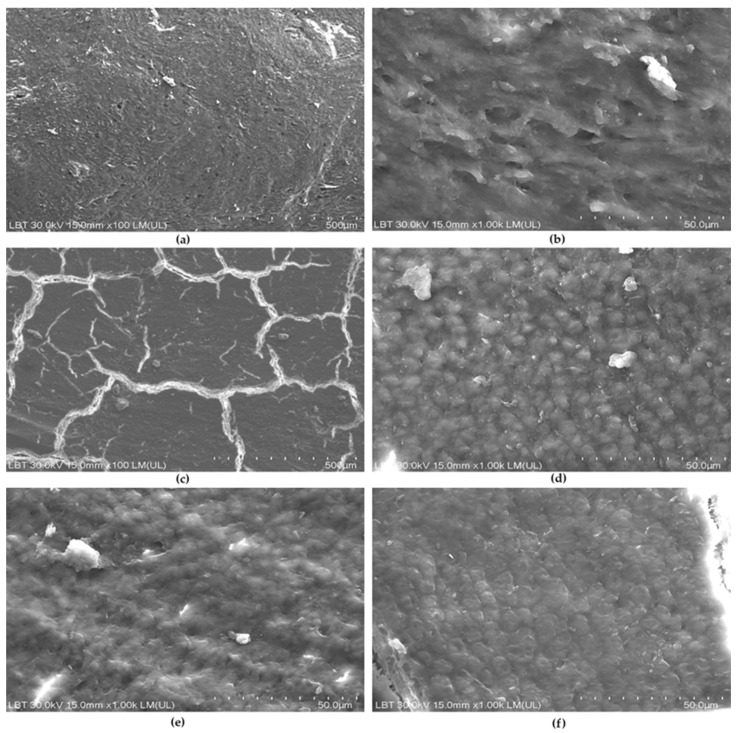
Instrumented samples: (**a**) SRP plus EDTA sample (S) with smear layer (×100, 500 µm bar). (**b**) SRP plus EDTA sample (S) with a smudged appearance (×1.00 k, 50 µm bar). (**c**) SRP plus erythritol plus EDTA sample (Se) with polygonal cracking (×100, 500 µm bar). (**d**) SRP plus erythritol plus EDTA sample (Se) with pebble-like microtexture (×1 k, 50 µm bar). (**e**) Discrete smear layer on a SRP plus erythritol plus EDTA sample (Se) (×1 k, 50 µm bar. (**f**) Discrete smear layer on a SRP plus glycine plus EDTA sample (Sg) (×1 k, 50 µm bar).

In the S group, the root surface appeared covered by a continuous smear layer, which largely obscured the underlying substrate (Figure 3a,b).

At ×100, the surface of Se samples exhibited pronounced polygonal cracking consistent with cementum, along with randomly distributed scratches (Figure 3c). At higher magnification, a cementum-like pebble microtexture was visible (Figure 3d), although it appeared slightly blurred in some samples, suggesting the presence of a discrete smear layer (Figure 3e). A similar appearance was observed in the Sg group (Figure 3f). No microislands of calculus remnants were observed on instrumented samples.

**Figure 4:**
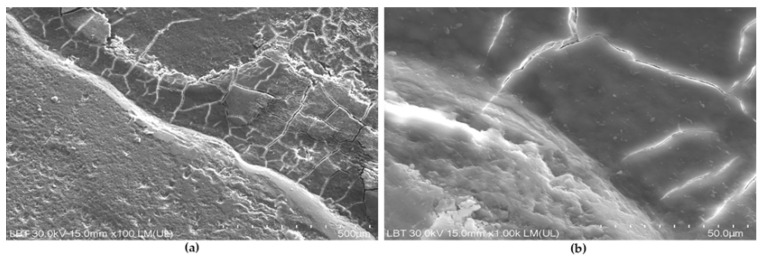
Instrumented sample from cement–enamel junction (JS): (**a**). Polygonal cracking of cementum in the upper right part, net demarcation line and enamel in the lower left part (×100, 500 µm bar). (**b**) Partially obscured cementum by residual deposits (×300, 100 µm bar).

SEM images of JS samples showed a clear demarcation line separating two morphologically distinct tissues. These samples exhibited polygonal cracking and a pebble-like surface consistent with cementum, partially obscured by a smear layer (Figure 4a,b). The adjacent region appeared smooth and compact at lower magnification, compatible with enamel-like characteristics.

Marks, cracks and smear layer scores are available in Table 2.

A statistically significant difference in the mark score was observed among S, Se, and Sg samples (Kruskal–Wallis H = 13.411, df = 2, *p* = 0.001). Bonferroni-adjusted pairwise comparisons showed that Se samples had significantly lower mark scores than S samples (*p* = 0.001). Differences between Sg and S samples and between Se and Sg samples did not reach statistical significance after Bonferroni correction (*p* = 0.054 and *p* = 0.646, respectively).

A statistically significant difference in the crack score was observed among S, Se, and Sg samples (Kruskal–Wallis H = 12.038, df = 2, *p* = 0.002). A significant difference was found between Se and S samples (*p* = 0.001), whereas the differences between Sg and S and between Se and Sg were not statistically significant (*p* = 0.314 and *p* = 0.018, respectively).

No statistically significant differences were observed among S, Se, and Sg samples for the smear layer score (Kruskal–Wallis H = 0.102, df = 2, *p* = 0.950).

The Mann–Whitney comparison between S and Js samples showed a statistically significant difference only for the mark score (U = 9.000, Z = −2.604, *p* = 0.009). No statistically significant differences were found for the crack score (*p* = 0.116) or the smear layer score (*p* = 0.913).

## 4. Discussion

The primary aim of the present study was to evaluate the microscopic effects of three treatment approaches on periodontitis-affected human root surfaces, focusing on mechanical alterations (marks and cracks) and smear layer presence (detritus removal).

SEM observations of control samples confirmed the presence of mature calculus deposits and remnants of EPSs, supporting the biological complexity of untreated periodontitis-affected root surfaces. The persistence of EPS-like structures may reflect the cohesive properties of the biofilm matrix and its relative resistance to specimen preparation procedures [50,51].

Cementum appeared to be preserved in the treated samples, as suggested by the characteristic pebble-like surface morphology and crack pattern. This finding is relevant because excessive instrumentation, mostly through hand curettes, may contribute to unnecessary root substance loss [52], whereas conservative surface management may better support periodontal healing [53,54]. In our samples, no residual calculus deposits were identified after treatment, suggesting effective debridement under the present experimental conditions. This is consistent with other *in vitro* findings reporting low median calculus scores after SRP [55]. In contrast, Cobb et al. reported residual calculus and biofilm after adjunctive EDTA application, with altered bacteria and a depleted extracellular matrix [11]. Similarly, SRP plus EDTA has been associated with 14–18% residual calculus compared with 45–53% after SRP alone [11].

Mechanical damage was assessed using a marks score intended to reflect operator- and device-related surface alterations rather than intrinsic substrate irregularities. The lower mark score observed in the Se group may suggest a less aggressive surface effect; however, a partial masking influence of residual smear layer cannot be excluded.

JS samples were included to explore instrumentation behavior in the clinically sensitive CEJ region. Although marks differed between S and Js samples, this comparison should be interpreted cautiously, since CEJ surfaces differ morphologically from mid-root areas and may respond differently to instrumentation independently of the applied protocol.

Previous studies have shown that different instrumentation methods produce distinct micromorphological patterns, ranging from grooves and deep marks after hand instrumentation [19,47,56], root loss after air-polishing [57,58] and increased roughness [47,59] after hand curettes to smoother or more homogeneous surfaces after selected ultrasonic or air-polishing approaches [55]. Together, these findings support the interpretation that the type and intensity of instrumentation may substantially influence surface preservation and the potential for subsequent biofilm retention.

Previous studies have long documented root surface alterations following periodontal instrumentation as promoters of biofilm development and continue to serve as essential reference points in this field [60,61]. Increased surface roughness promotes bacterial adhesion and plaque formation [60]; moreover, an increase in hard-tissue roughness from the accepted threshold of 0.2 μm to 0.8 μm significantly enhanced bacterial adhesion and biofilm formation after SRP [61].

Cracks were consistently associated with cementum and are likely attributable to dehydration artifacts during specimen processing. Their identification was useful in distinguishing cementum from calculus and dentin, as the latter did not display comparable crack patterns [55]. Evidence that routine periodontal scaling alone produces persistent microcracks independent of SEM preparation remains limited [62,63], although cracks after hand instrumentation have been reported [56]. After Five and Mini Five curettes have been shown to induce gouging and cracks of varying sizes compared with conventional Gracey curettes [19]. Curettes produced a thick multilayered smear layer, ultrasonic instrumentation produced a thinner smear layer with few grooves, and the combination resulted in slender smear layer remnants with occasional exposed collagen fibrils [64].

Smear layer formation after mechanical instrumentation has received considerable attention. In the present study, no statistically significant differences in smear layer scores were observed among treatment approaches; however, further research is required to better elucidate this aspect.

Smear layer formation remains clinically relevant because it may act as a contaminated layer between the treated root surface and the healing periodontal tissues [65]. Smear layer thickness depends on instrumentation method, adjunctive agents [19,28,29,33], instrument sharpness and type [66], with reported values ranging from 2 to 15 μm [67]. In the present study, smear layer scores did not differ significantly among treatment protocols, suggesting that the adjunctive use of EDTA and air-polishing powders did not clearly modify this parameter under the present conditions. This may reflect the strong adherence of the smear layer to root surfaces [67] and the multifactorial nature of its formation [19,28,29,66]. Although EDTA has been described as capable of exposing patent dentinal tubules as a marker of smear layer removal [68,69] dentinal tubules were not identified in the SEM areas assessed in the present study. Nevertheless, EDTA remains clinically attractive due to its near-neutral pH, which may limit cytotoxicity and improve periodontal cell compatibility [42,47].

However, a physiological elimination of the smear layer produced on root surfaces after SRP is expected in vivo following a biphasic pattern, with a rapid reduction within 7 days and a slow lost driving to a significant decrease 28 days after SRP [70].

A limitation of the present study is the relatively small number of teeth included per treatment group, which may limit the generalizability of the findings. A further limitation is the absence of formal intra- or inter-examiner calibration, although evaluations were performed by experienced investigators using predefined criteria.

Another limitation is related to the ex vivo design and SEM-based assessment. Although SEM provides detailed morphological information, specimen preparation procedures (fixation, dehydration, coating) may influence surface appearance, and the semi-quantitative scoring approach cannot fully reflect three-dimensional surface alterations and is related to a certain degree of subjectivity. However, efforts were made to increase the clinical relevance of the experimental model by using periodontitis-affected human roots with calculus deposits, rather than bovine teeth [47] or teeth from periodontally healthy donors. In addition, samples were rinsed using a dental unit water spray instead of an ultrasonic bath [71], air polishing was performed at a standardized 5 mm working distance [47,48] for 5 s [72] in accordance with published data, and instrumentation combined ultrasonic and hand devices as recommended in current clinical guidelines [3,4].

Cementum appeared to be preserved in all treated groups, suggesting that the applied protocols may be compatible with conservative instrumentation under the present experimental conditions. Achieving a decontaminated, minimally damaged, and biologically acceptable cemental surface through SRP is important for optimal healing, improved clinical outcomes [7] including connective tissue attachment gain [73,74].

Although the SEM findings provided a clear depiction of root surface morphology, their interpretation should be approached with caution due to methodological limitations, including the limited number of biological samples and examined fields, the in vitro nature of the study, and the fact that multiple samples were obtained from the same tooth, which may introduce a degree of dependence between observations.

## 5. Conclusions

EDTA did not result in complete smear layer removal under the tested conditions; however, its potential role in reducing subgingival deposits supports its role as a conservative adjunctive in periodontal therapy.

Root surfaces at the CEJ exhibited a similar response to instrumentation as mid-root areas, indicating that controlled mechanical debridement can be performed in this anatomically challenging region without inducing additional surface damage.

## Figures and Tables

**Table 1:** Coding system for SEM-examined root samples based on applied treatments.

Label	Sample Type and Treatment
**Ct**	Controls
**S**	Middle root sample, SRP + EDTA
**Sg**	Middle root sample, SRP + AIR-FLOW^®^ PERIO Powder + EDTA
**Se**	Middle root sample, SRP + AIR-FLOW^®^ PLUS Powder + EDTA
**JS**	CEJ samples, SRP + EDTA

CEJ, cementoenamel junction; EDTA, ethylenediaminetetraacetic acid; SRP, scaling and root planing.

**Table 2:** SEM scores (0–3) expressed as median (25th–75th percentile).

Variable (0–3)	S	Se	Sg	Js
**Mark score (*n* = 8)**	3.00 (2.25–3.00)	1.00 (1.00–1.75)	2.00 (1.00–2.00)	2.00 (1.00–2.00)
**Crack score (*n* = 8)**	2.00 (1.00–2.00)	3.00 (3.00–3.00)	2.00 (1.25–2.75)	2.50 (1.25–3.00)
**Smear layer score (*n* = 8)**	1.00 (0.25–2.00)	1.00 (0.00–2.00)	1.00 (0.25–2.00)	1.00 (0.00–2.75)

S, scaling and root planing (SRP) + ethylenediaminetetraacetic acid (EDTA) sample; Se, SRP + erythritol + EDTA sample; Sg, SRP + glycine + EDTA sample; Js, cementoenamel junction (CEJ) sample treated by SRP + EDTA.

## Data Availability

The data are available from the corresponding author upon reasonable request.
